# Impact of Real‐Time, On‐Demand Influenza and Respiratory Syncytial Virus Testing at Point‐of‐Care on Antibiotic Prescribing and Clinical Outcome in Pediatric Outpatients With Acute Respiratory Illness: A Prospective, Quasi‐Randomized, Controlled Study

**DOI:** 10.1111/irv.70205

**Published:** 2025-12-31

**Authors:** Yue Xie, Tianming Chen, Bing Liu, Haijuan Xiao, Xinghui Gao, Qinjing Li, Bing Hu, Cuiying Liu, Chengsong Zhao, Yuchuan Li, Xin Xu, Mengran Li, Yi‐Wei Tang, Gang Liu

**Affiliations:** ^1^ Department of Infectious Diseases, Key Laboratory of Major Diseases in Children, Ministry of Education Beijing Children's Hospital, Capital Medical University, National Center for Children's Health Beijing China; ^2^ Research Unit of Critical Infection in Children Chinese Academy of Medical Sciences, 2019RU016 Beijing China; ^3^ Medical Affairs, Danaher Diagnostic Platform/Cepheid Shanghai China; ^4^ Beijing Children's Hospital, Capital Medical University, National Center for Children's Health Beijing China; ^5^ Outpatient Department Beijing Children's Hospital, Capital Medical University, National Center for Children's Health Beijing China; ^6^ Department of Information Center Beijing Children's Hospital, Capital Medical University, National Center for Children's Health Beijing China; ^7^ Department of Biostatistics & Data Management Beckman Coulter China, Danaher Shanghai China; ^8^ College of Public Health Chongqing Medical University Chongqing China

**Keywords:** antibiotic prescription, influenza viruses, point‐of‐care testing, real‐time clinical management, respiratory syncytial virus

## Abstract

**Background:**

Rapid and accurate detection and identification of viral pathogens have an impact on physician decision‐making for patients with acute respiratory illness (ARI). We aimed to evaluate the Xpert Xpress Flu/RSV test for the management of antibiotic prescribing in pediatric outpatients with ARI.

**Methods:**

We performed a prospective, quasi‐randomized, controlled study in Beijing Children's Hospital between December 1, 2021 and April 28, 2022. Outpatients with ARI aged 28 days to 18 years were enrolled and randomly assigned to the Xpert Xpress Flu/RSV test (Xpert) group or the influenza (Flu) antigen test (control) group. Both tests were performed on site.

**Results:**

A total of 771 patients were enrolled and assigned randomly to the Xpert (*n* = 398) and the control (*n* = 373) groups. There was no statistically significant difference in antibiotic prescriptions between the two groups, whereas a significant difference was observed for the prescriptions of oseltamivir (*p* < 0.001). In Flu B‐positive patients, a statistically significant decrease in antibiotic use and increase in antiviral use were observed in both Xpert and control groups. Cephalosporin use was significantly decreased in respiratory syncytial virus (RSV)‐positive patients in the Xpert group before (*n* = 8, 17.4%) and after (*n* = 1, 2.2%)visit (*p* = 0.035). Among clinical and laboratory parameters, shorter fever length (OR = 0.366) and positive Flu B or RSV (OR = 3.99) were two independent factors for antibiotic withdrawal by logistic regression analysis. There was no significant difference in duration of fever, clinical outcomes, and expenditure between the two groups at the 7‐day and 30‐day follow‐up.

**Conclusions:**

Use of Xpert Xpress Flu/RSV at point‐of‐care in pediatric outpatients with ARI reduced antibiotic prescription, which has the potential to improve antibiotic stewardship.

## 1 Introduction

Acute respiratory illness (ARI) is one of the leading causes of outpatient visits for children worldwide and it is mainly caused by viruses, such as respiratory syncytial virus (RSV), influenza (Flu), parainfluenza virus, and human rhinovirus [1, 2]. However, children with ARI are likely to receive an antibiotic prescription. In France, 44.7% of respiratory tract infections (RTIs) that were presumed viral infections still received antibiotics [3]. In the United States, 28.1% to 44.4% of children in ambulatory care and 55% to 57% of children in pediatric emergency departments and urgent care settings are prescribed antibiotics [2, 4, 5]. Antibiotic prescribing is particularly common in China, with antibiotics prescribed in over 70% of children with upper RTIs, which are usually caused by viruses [6]. Inappropriate use of antibiotics can lead to drug‐related side effects, the development of antibiotic resistance, and increased health care costs [7].

Rapid and accurate detection of viruses can contribute to a reduction in antibiotic prescriptions, appropriate antiviral use, and improved prognosis [8, 9]. Antigen‐based tests are widely used for influenza detection in China but have lower sensitivity than molecular methods. The Xpert Xpress Flu/RSV test (Cepheid, Sunnyvale, CA, USA) is a rapid, random‐access test that enables simultaneous multiplex testing for Flu A, Flu B, and RSV in upper respiratory tract specimens with higher sensitivity and specificity than traditional antigen‐based methods [10].

Although the findings on whether antigen‐based tests for influenza can reduce antibiotic use are not entirely consistent [11], the majority of studies suggest that it could attribute to the rational use of antibiotics for ARI [12, 13], whereas data on the impact of Xpert Xpress Flu/RSV testing in children are limited. We performed a prospective, quasi‐randomized, controlled study on ARI pediatric outpatients to evaluate whether the use of the Xpert Xpress Flu/RSV test would reduce unnecessary antibiotic use.

## 2 Methods

### 2.1 Study Population

We performed a prospective, quasi‐randomized, controlled study in Beijing Children's Hospital (the National Center for Children's Health in China), a large tertiary hospital with 970 beds in Beijing, China. From December 1, 2021 to April 28, 2022, consecutive patients aged 28 days to 18 years presenting to the outpatient department of Beijing Children's Hospital were enrolled if they met the following criteria: (1) duration of fever ≤ 7 days with axillary temperature ≥ 38°C; (2) clinical symptoms and/or signs of respiratory infection, such as cough or rhinorrhea; and (3) C‐reactive protein (CRP) < 40 mg/L. Children were excluded if they had (1) confirmed viral respiratory infection before visit to our hospital; (2) definite or probable bacterial infection, including urinary tract infection, skin and soft tissue infection, bone or joint infection, or bacteremia; or (3) definite or probable infectious diseases caused by other organisms, such as mycoplasma, chlamydia, or pneumocystis.

### 2.2 Study Design

All enrolled patients were subdivided according to the date of visit, assigned to the Xpert group on odd‐numbered days and the control group on even‐numbered days, treated by two senior pediatricians who had the same clinical experience in pediatric infectious diseases, and received a standard clinical assessment. Nasopharyngeal swabs were obtained, and tests were immediately performed by doctors who were board‐certified in pediatrics. Patients in the Xpert group were tested with the Xpert Xpress Flu/RSV (Cepheid, Sunnyvale, CA, USA) test and a Flu antigen detection via lateral flow test (Genesis, Hangzhou, China) at the same time, whereas in the control group only a Flu antigen test was performed. The two tests were performed in the mini‐lab within the outpatient department following the instructions of the manufacturer. The results were available within 32 min for the Xpert Xpress Flu/RSV test and 15 min for the Flu antigen test. In addition, complete blood count and severe acute respiratory syndrome coronavirus 2 (SARS‐CoV‐2) tests (DAAN GENE, Guangzhou, China) were performed on all patients, while procalcitonin, CRP, and chest X‐ray were done depending on the patients' condition.

The primary outcome measure was receipt of an antibiotic and anti‐influenza drug prescription on the day of enrollment. Secondary endpoints were the positive rate of virus detection, duration of fever after visit, clinical outcomes, and cost of medical care. In addition, we also analyzed the factors contributing to antibiotic withdrawal among all enrolled patients. The definition of antibiotic withdrawal: Antibiotics were taken before this visit (patient took antibiotics without doctor's advice or sought medical care prior to the visit) and discontinued after seeking medical care to our hospital.

Antibiotics were classified by the World Health Organization (WHO) into four groups: Access, Watch, Reserve (AWaRe), and Not Recommended group [14]. Access antibiotics have a narrow spectrum of activity, lower cost, a good safety profile, and generally low resistance potential. Watch antibiotics are broader‐spectrum antibiotics, generally with higher costs, and are recommended only as first‐choice options for patients with more severe clinical presentations or for infections where the causative pathogens are more likely to be resistant to Access antibiotics. Reserve antibiotics are last‐choice antibiotics used to treat multidrug‐resistant infections.

The study protocol was registered with Chinese Clinical Trials (ChiCTR2500110231). Informed consent was obtained from all participants or their guardians prior to enrollment.

### 2.3 Data Collection

Demographic information, clinical presentation and exam findings, chest X‐ray results, laboratory test results, medication use, expense, and randomization status of each patient were recorded on the case report form for further analysis. After the visit, a telephone follow‐up was conducted on Days 7 and 30 by the same pediatrician who saw the patient to collect information on duration of fever, clinical outcome, and adverse drug reactions.

### 2.4 Statistical Analysis

Categorical variables were presented as numbers and percentages, while continuous variables were shown as the median and interquartile range (IQR). Categorical variables were compared using the Chi‐square or Fisher's exact tests as appropriate. Continuous variables within two groups were compared using *t*‐test or Mann–Whitney *U* test according to their distribution. Univariable logistic regression was used to evaluate the factors associated with the probability of withdrawing antibiotics; when considering factors with *p* < 0.1, multivariable logistic regression was made. The odds ratio (OR) and confidence interval at 95% (95% CI) were presented. *p*‐value < 0.05 was considered significant. All of the statistical analyses were conducted using Statistical Product and Service Solutions (SPSS), version 20.0 (IBM, NY, USA).

## 3 Results

### 3.1 Clinical Characteristics

**FIGURE 1:**
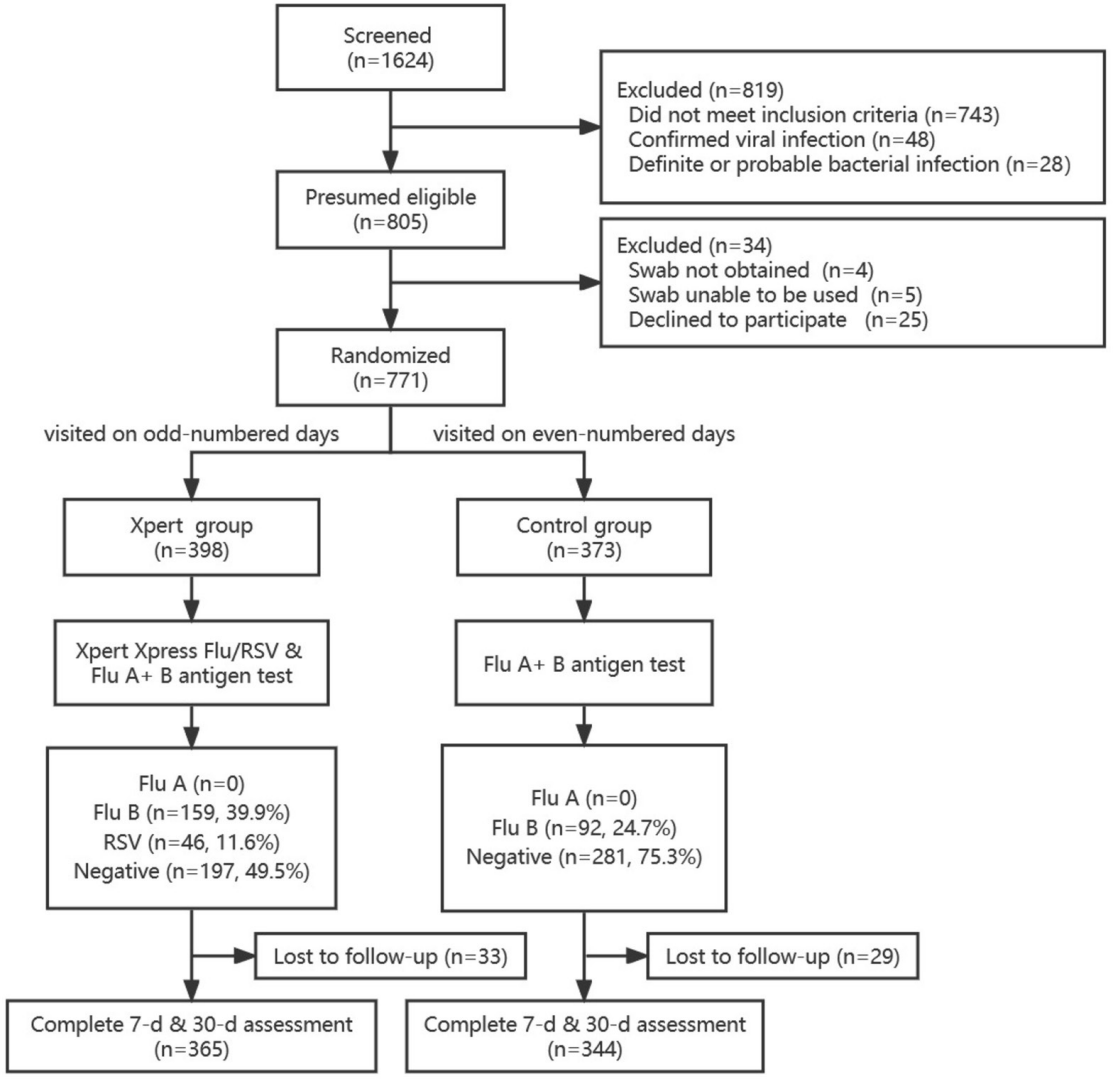
The flow chart of outpatients with ARI for enrollment, virus detection, and follow‐up. Flu = influenza, RSV = respiratory syncytial virus.

Between December 1, 2021 and April 28, 2022, 771 ARI outpatients were enrolled and assigned randomly into the Xpert group (*n* = 398) or the control (*n* = 373) group (Figure 1). The median age was 4.3 years (IQR 2.9–6.1), 456 (59.1%) of patients were under 5 years of age and 410 (53.2%) were male. The overall median duration of fever was 3.5 days (IQR 3.0–5.0), and the median duration of fever was 2 days (IQR 1–3) following the hospital visit. During the course of illness, 175 (22.7%) visited other hospitals before this visit. A comparison of clinical features was listed in Table 1.

**TABLE 1:** Comparison of clinical features between the two groups.

	Xpert group (*n* = 398)^a^	Control group (*n* = 373)^a^	*p*
Male gender	213 (53.5)	197 (52.8)	0.845
Age in years (median, IQR)	4.4 (2.8–6.4)	4.3 (3.0–5.8)	0.509
Clinical presentations			
Days of fever (median, IQR)	2 (1–3)	1 (1–3)	0.537
T > 39°C	184 (46.2)	183 (49.1)	0.432
Cough	26 (66.8)	244 (65.4)	0.677
Rhinorrhea	143 (35.9)	142 (38.1)	0.538
Sore throat	122 (30.7)	98 (26.3)	0.551
Abdominal pain	26 (6.5)	16 (4.3)	0.170
Vomiting	18 (4.5)	8 (2.1)	0.068
Diarrhea	7 (1.8)	13 (3.5)	0.132
Headache	12 (3.0)	6 (1.6)	0.198
X‐ray indicated pneumonia	23/45 (51.1)	21/50 (42.0)	0.374
Laboratory results			
WBC (×10^9^/L) (median, IQR)	6.0 (4.4–8.1)	6.4 (4.7–9.1)	0.042
Neutrophils (×10^9^/L) (median, IQR)	3.1 (2.0–5.1)	3.7 (2.3–5.7)	0.013
CRP ≥ 8 mg/L	118 (29.6)	120 (32.2)	0.665
PCT > 0.1 ng/mL	55/271 (20.3)	46/232 (19.8)	0.896
First visit	307 (77.1)	282 (75.6)	0.617

Abbreviations: CRP = C‐reactive protein, PCT = procalcitonin, WBC = white blood cell.

^a^ *n* (%).

### 3.2 Virus Detection

Flu B was detected in 251 (32.6%) patients. Neither Flu A nor SARS‐CoV‐2 (due to the prevention and control of the epidemic, positive of SARS‐CoV‐2 was isolated and treated in the specific institution) was detected during the study period. Flu B and RSV were detected in 159 (39.9%) and 46 (11.6%) patients in the Xpert group, respectively (Table 2). There were four cases that were positive for both Flu B and RSV. Flu B was detected in 92 (24.7%) patients in the control group. The positive rate of Flu B was higher in the Xpert group than the control group (*p* < 0.001). In 30 (7.5%) cases, Flu B was detected by the Xpert test, but the Flu B antigen test was negative (antigen test‐false‐negative rate 18.9%).

**TABLE 2:** Comparison of pathogen detection, management, follow‐up, and expense between two groups.

	Xpert group (*n* = 398)^a^	Control group (*n* = 373)^a^	*p*
Pathogen detection			
Flu B	159 (39.9)	92 (24.7)	< 0.001
RSV	46 (11.6)	—	—
Prescriptions on the day of enrollment			
Intravenous antibiotics	22 (5.5)	18 (4.8)	0.661
Oral antibiotics	57 (14.3)	68 (18.2)	0.141
Macrolides	49 (12.3)	52 (13.9)	0.503
Cephalosporins	19 (4.8)	20 (5.4)	0.710
Clindamycin	10 (2.5)	9 (2.4)	0.929
Faropenem	4 (1.0)	2 (0.5)	0.741
Total antibiotic use	79 (19.8)	86 (23.1)	0.278
Oseltamivir	155 (38.9)	89 (23.9)	< 0.001
Peramivir	8 (2.0)	6 (1.6)	0.677
Total anti‐influenza drug use^b^	157 (39.4)	90 (24.1)	< 0.001
Day 7 assessment (*n* = 709)			
Duration of fever after visit (median, IQR)	2 (1–2)	2 (1–2)	0.593
Recovery	207/365 (56.7)	185/344 (53.8)	0.432
Cough	141/365 (38.6)	131/344 (38.1)	0.929
Rhinorrhea	56/365 (15.3)	57/344 (16.6)	0.655
Day 30 assessment (*n* = 709)			
Hospitalization	1/365^c^ (0.3)	0 (0)	1.000
Expense (yuan)			
Drug cost (median, IQR)	420 (256–559)	408 (263–550)	0.749
Total cost (median, IQR)	450 (289–628)	430 (300–610)	0.739

Abbreviations: Flu = influenza, RSV = respiratory syncytial virus.

^a^ *n* (%).

^b^ Including standard treatment with oseltamivir or peramivir.

^c^ One patient hospitalized due to pneumonia.

### 3.3 Antibiotic Use

Antibiotics were prescribed to 165 (21.4%) children after their visit. Among them, 125 (16.2%) were given oral antibiotics, and 40 (5.2%) patients were administered antibiotics intravenously. In patients who received antibiotic therapy, 11.5% of patients (*n* = 19) received “Access” antibiotics (clindamycin), and 88.5% of patients (*n* = 146) received “Watch” antibiotics, including macrolides (101, 61.2%), cephalosporins (39, 23.6%), clindamycin (19, 11.5%), and faropenem (6, 3.6%).

In the Xpert group, the total antibiotic use rate was 19.8%; 14.3% were prescribed by oral and 5.5% by intravenous. Macrolides (12.3%) were the most frequently prescribed class of antibiotics, followed by cephalosporins (4.8%), clindamycin (2.5%), and faropenem (1.0%). In the control group, the antibiotic prescription rate was 23.1%, with oral and intravenous administration accounting for 18.2% and 4.8%, respectively. In terms of drug classes, macrolides constituted the largest share of prescriptions (13.9%), followed by cephalosporins (5.4%), clindamycin (2.5%), and faropenem (1.0%). The route of administration and type of antibiotic prescribed showed no difference between two groups (Table 3).

**TABLE 3:** Antibiotic and anti‐influenza drug use in two groups before and after visit.

	Xpert group (*n* = 398)^a^			Control group (*n* = 373)^a^		
	Before visit	After visit	*p*	Before visit	After visit	*p*
Intravenous antibiotics	14 (3.5)	22 (5.5)	0.170	14 (3.8)	18 (4.8)	0.470
Oral antibiotics	70 (17.6)	57 (14.3)	0.208	35 (9.4)	68 (18.2)	< 0.001
Macrolides	55 (13.8)	49 (12.3)	0.528	25 (6.7)	52 (13.9)	0.001
Cephalosporins	37 (9.3)	19 (4.8)	0.013	24 (6.4)	20 (5.4)	0.534
Clindamycin	1 (0.3)	10 (2.5)	0.006	3 (0.8)	9 (2.4)	0.081
Faropenem	0 (0)	4 (1.0)	0.133	0 (0)	2 (0.5)	0.479
Total antibiotic use	84 (21.1)	79 (19.8)	0.661	49 (13.1)	86 (23.1)	< 0.001
Oseltamivir	6 (1.5)	155 (38.9)	< 0.001	0 (0)	89 (23.9)	< 0.001
Peramivir	0 (0)	8 (2.0)	0.013	0 (0)	6 (1.6)	0.040
Total anti‐influenza drug use	6 (1.5)	157 (39.4)	< 0.001	0 (0)	90 (24.1)	< 0.001

^a^ *n* (%).

In the Xpert group, cephalosporin use decreased from 9.3% before the visit to 4.8% (*p* = 0.013) after the visit. However, in the control group, only cephalosporin use was modestly reduced by 1% after the hospital visit, whereas the use of other antibiotic classes increased. Moreover, in both the Xpert and control groups, antibiotic use was significantly decreased in Flu B‐positive patients (Table 4). In the Xpert group, overall antibiotic use was reduced from 22.6% to 5.7% after the hospital visit (*p* < 0.001), and oral antibiotic use was reduced from 20.8% to 3.8% (*p* < 0.001). Macrolide use was reduced from 15.7% to 5.0% (*p* = 0.002), and cephalosporin use was reduced from 9.4% to 0% (*p* < 0.001). In the control group, the percentage of all antibiotics (13.0% vs. 3.3%, *p* = 0.015), intravenous antibiotics (3.3% vs. 0%, *p* = 0.244), oral antibiotics (9.8% vs. 3.3%, *p* = 0.073), macrolides (6.5% vs. 3.3%, *p =* 0.494), and cephalosporins (5.4% vs. 0%, *p* = 0.070) decreased after the hospital visit.

**TABLE 4:** Antibiotic and anti‐influenza drug use among Flu B‐positive and Flu B‐negative patients in two groups before and after visit.

	Xpert group (*n* = 398)^a^						Control group (*n* = 373)^a^						*p* ^b^	*p* ^c^
	Flu B‐positive (*n* = 159)			Flu B‐negative (*n* = 239)			Flu B‐positive(*n* = 92)			Flu B‐negative(*n* = 281)				
	Before visit	After visit	*p*	Before visit	After visit	*p*	Before visit	After visit	*p*	Before visit	After visit	*p*		
Intravenous antibiotics	3 (1.9)	3 (1.9)	1.000	11 (4.6)	19 (7.9)	0.131	3 (3.3)	0 (0)	0.244	11 (3.9)	18 (6.4)	0.182	0.010	0.470
Oral antibiotics	33 (20.8)	6 (3.8)	< 0.001	37 (15.5)	51 (21.3)	0.098	9 (9.8)	3 (3.3)	0.073	26 (9.3)	65 (23.1)	< 0.001	< 0.001	1.000
Macrolides	25 (15.7)	8 (5.0)	0.002	30 (12.6)	41 (17.2)	0.157	6 (6.5)	3 (3.3)	0.494	19 (6.8)	49 (17.4)	< 0.001	< 0.001	0.734
Cephalosporins	15 (9.4)	0 (0)	< 0.001	22 (9.2)	19 (7.9)	0.624	5 (5.4)	0 (0)	0.070	19 (6.8)	20 (7.1)	0.868	< 0.001	—
Clindamycin	0 (0)	1 (0.6)	1.000	1 (0.4)	9 (3.8)	0.011	1 (1.1)	0 (0)	1.000	2 (0.7)	9 (3.2)	0.033	0.103	1.000
Faropenem	0 (0)	0 (0)	—	0 (0)	4 (1.8)	0.132	0 (0)	0 (0)	—	0 (0)	2 (0.7)	0.479	0.260	—
Total antibiotic use	36 (22.6)	9 (5.7)	< 0.001	48 (20.1)	70 (29.3)	0.020	12 (13.0)	3 (3.3)	0.015	37 (13.2)	83 (29.5)	< 0.001	< 0.001	0.581
Oseltamivir	4 (2.5)	154 (96.9)	< 0.001	2 (0.8)	1 (0.4)	1.000	0 (0)	89 (96.7)	< 0.001	0 (0)	0 (0)	—	< 0.001	1.000
Peramivir	0 (0)	8 (5.0)	0.012	0 (0)	0 (0)	—	0 (0)	6 (6.5)	0.038	0 (0)	0 (0)	—	0.002	0.620
Total anti‐influenza drug use	4 (2.5)	156 (98.1)	< 0.001	2 (0.8)	1 (0.4)	1.000	0 (0)	90 (97.8)	< 0.001	0 (0)	0 (0)	—	< 0.001	1.000

^a^ *n* (%).

^b^ Flu B‐positive after visit vs. Flu B‐negative after visit in the Xpert group.

^c^ Flu B‐positive after visit in the Xpert group vs. Flu B‐positive after visit in the Control group.

Cephalosporin use was reduced in RSV‐positive patients tested with the Xpert test (17.4% vs. 2.2%, *p* = 0.035) (Table 5). However, in the Xpert group, total antibiotic, oral antibiotic, macrolides, and clindamycin use was higher in RSV‐positive patients compared with RSV‐negative patients after the hospital visit.

**TABLE 5:** Antibiotic use among patients of RSV‐positive and RSV‐negative in the Xpert group before and after visit.

	RSV‐positive (*n* = 46)^a^			RSV‐negative (*n* = 352)^a^			*p* ^b^
	Before visit	After visit	*p*	Before visit	After visit	*p*	
Intravenous antibiotics	6 (13.0)	4 (8.7)	0.503	8 (2.3)	18 (5.1)	0.046	0.511
Oral antibiotics	14 (30.4)	19 (41.3)	0.277	56 (15.9)	38 (10.8)	0.046	< 0.001
Macrolides	14 (30.4)	15 (32.6)	0.822	41 (11.6)	34 (9.7)	0.392	< 0.001
Cephalosporins	8 (17.4)	1 (2.2)	0.035	29 (8.2)	18 (5.1)	0.097	0.609
Clindamycin	0 (0)	6 (13.0)	0.035	1 (0.3)	4 (1.1)	0.368	< 0.001
Faropenem	0 (0)	1 (2.2)	1.000	0 (0)	3 (0.9)	0.247	0.389
Total antibiotic use	20 (43.5)	23 (50.0)	0.531	64 (18.2)	56 (15.9)	0.423	< 0.001

Abbreviation: RSV = respiratory syncytial virus.

^a^ *n* (%).

^b^ RSV‐positive after visit vs. RSV‐negative after visit.

Prior to the hospital visit, 132 (17.1%) patients were treated with antibiotics. Within this group, 71 (9.2%) received antibiotics without any medical advice (parents decided to administer antibiotics to their children). Antibiotics were withdrawn in 60 (45.5%) of 132 patients after the hospital visit. On univariable analysis, antibiotic discontinuation was more frequently observed in older children with shorter duration of fever prior to the visit, a normal chest X‐ray, and lower white blood cell count, neutrophil count, and CRP (*p* < 0.001). On multivariable analysis, shorter fever duration (OR 0.366 [0.239–0.560], *p* < 0.001) and a positive Flu B or RSV test (OR 3.990 [1.330–11.972], *p* = 0.0136) were two independent factors that led to antibiotic discontinuation (Table 6).

**TABLE 6:** Univariable and multivariable logistic regression to evaluate the risk factors of probability of antibiotics withdrawal.

	Withdrawal (*n* = 60)^a^	Continued (*n* = 72)^a^	*p*	Multivariable	
				OR (95% CI)	*p*
Group			0.411		
Xpert	40 (66.7)	43 (59.7)			
Control	20 (33.3)	29 (40.3)			
Male gender	26 (43.3)	38 (52.8)	0.280		
Age in years (median, IQR)	5.0 (3.8–7.6)	3.6 (2.5–5.3)	< 0.001	1.17 (0.942–1.453)	0.157
Clinical presentations					
Days of fever (median, IQR)	2.0 (1.0–3.0)	4.0 (3.0–5.5)	< 0.001	0.366 (0.239–0.560)	< 0.001
Temperature	39.3 (39.0–39.6)	39.2 (39.0–39.6)	0.872		
Laboratory results					
WBC (×10^9^/L) (median, IQR)	5.4 (3.4–7.5)	8.1 (5.7–9.6)	< 0.001	0.874 (0.742–1.030)	0.109
Neutrophils (×10^9^/L) (median, IQR)	2.9 (1.5–4.7)	3.8 (2.2–6.4)	0.026	—	—
CRP < 8 mg/L	51 (85.0)	38 (52.8)	< 0.001	0.470 (0.130–1.695)	0.249
PCT < 0.5 ng/mL	34 (56.7)	33 (45.8)	0.215		
Flu B or RSV positive	45 (75.0)	21 (29.2)	< 0.001	3.990 (1.330–11.972)	0.014
X‐ray indicated pneumonia	0 (0)	21/38 (55.3)	< 0.001	—	—

Abbreviations: CRP = C‐reactive protein, Flu = influenza, PCT = procalcitonin, RSV = respiratory syncytial virus, WBC = white blood cell.

^a^ *n* (%).

### 3.4 Anti‐Influenza Therapy

Anti‐influenza treatments were given to 247 (32.0%) patients after the visit. Among them, 233 (30.2%) were administered oseltamivir, 3 (0.4%) received peramivir, and 11 (1.4%) were initially treated with peramivir and then followed by oseltamivir. Six patients had already taken oseltamivir without detection of flu before visit to our hospital. One patient who had influenza‐like symptoms but tested negative for influenza was empirically prescribed oseltamivir.

In both Xpert and control groups, a statistically significant antiviral use increase was observed in Flu B‐positive patients (Table 4). However, more patients received oseltamivir in the Xpert group after the hospital visit (38.9% vs. 23.9%, *p* < 0.001). There was no significant difference in peramivir prescriptions between the two groups after the visit.

### 3.5 Expense and Follow‐Up

Finally, the median drug cost was 420 (IQR 256–559) yuan in the Xpert group and 408 (IQR 263–550) yuan in the control group (*p* = 0.749). And the total median cost was 450 (IQR 289–628) yuan and 430 (IQR 300–610) for the Xpert group and control group, respectively (*p* = 0.739) (Table 2).

A total of 709 (92.0%) participants completed a telephone follow‐up survey on Days 7 and 30 after the hospital visit. On Day 30 after the visit, one patient in the Xpert group was admitted to hospital due to pneumonia. There were no deaths or drug‐related side effects reported in the two groups.

## 4 Discussion

In our study, we evaluated the impact of the Xpert Xpress Flu/RSV test on the clinical management of pediatric outpatients presenting with ARI. The findings of our study showed that the use of the Xpert test performed at the point‐of‐care could help to reduce the prescription of antibiotics.

Viruses are the most common causes of ARI in children seeking medical care [15]. In our study, nearly half of patients with ARI in the winter–spring season had viral infection, due to influenza B or RSV. However, it is often a challenge for clinicians to accurately distinguish viral ARI from bacterial infection [16]. Timely diagnosis is crucial because delays in appropriate management are associated with poor prognosis and overuse of antibiotics. Respiratory viral tests performed at the visit may promote the appropriate use of antibiotics and limit unnecessary prescriptions [17]. In our study, 132 (17.1%) children received antibiotic therapy without any pathogen detection before visit to our hospital. Among them, 66 (50.0%) were ultimately shown to have a viral infection. Utilization of antibiotics in these patients could have been avoided if the virus detection was performed earlier.

Rapid antigen tests (RATs) for influenza typically have low sensitivity, ranging from 42% to 64% according to published reports [18]. Our data showed a higher positive rate in detection for Flu B when using the Xpert test compared with the antigen test. In the Xpert group, the RAT‐false‐negative rate of the antigen test was 18.9% for detection of Flu B. Without utilization of the Xpert test, antiviral treatment may have been withheld in these patients, which could impact clinical outcome [19]. Studies have shown that when compared with other standard‐of‐care nucleic acid amplification tests, the Xpert test is a rapid, sensitive, and reliable test for detecting Flu A, Flu B, and RSV infections in pediatric patients [20, 21].

Current findings on whether rapid pathogen testing can reduce antibiotic use remain inconsistent [22, 23]; however, our study showed the use of Xpert test to identify respiratory viruses in children decreases unnecessary antibiotic use. In the Xpert group, cephalosporins use was significantly decreased after the hospital visit. For Flu B‐positive patients in the Xpert group, there was a decrease in the use of total antibiotics, oral antibiotics, and macrolides. Notably, no patients with Flu B in the Xpert group were prescribed cephalosporins. For RSV‐positive patients, cephalosporin use was also significantly reduced. The most commonly prescribed antibiotics in children with ARI were third‐generation cephalosporins [24, 25]. It is encouraging that our study showed the Xpert test was associated with a decrease in unnecessary prescriptions of cephalosporins. Unlike in other countries, China has the highest proportion of “Watch” antibiotic prescriptions, which include antibiotic classes with a higher resistance potential [26]. This is primarily due to the high prescription rates of cephalosporins. The point‐of‐care testing (POCT) appears to have reversed this trend, leading to a significant reduction in the proportion of “Watch” antibiotics, highlighting the effectiveness of POCT in improving antibiotic stewardship. The application of POCT to encourage the rational use of cephalosporins is worth fully assessing and promoting.

The overuse of antimicrobials and increasing rates of antimicrobial resistance in China is a serious concern. Previous studies showed that antimicrobial stewardship programs were crucial to promote reasonable administration of antibiotics for RTIs [27]. According to the AWaRe books published by WHO [14], antibiotics within the “Access group” are recommended as empiric options for ARI caused by bacteria. However, only 11.5% of antibiotics use in our study belonged to the “Access group,” which is consistent with reported antibiotic prescribing practices in China [28]. In addition, amoxicillin is the first choice for ARI, but in our study, physicians tended to prescribe second‐ or third‐generation cephalosporins for possible bacterial infections. Meanwhile, clindamycin use was increased in the Xpert group and particularly in the RSV‐positive patients, which could be partly due to the concern about bacterial co‐infection such as *Pneumococcus*. However, on the basis of surveillance of bacterial resistance in China, *Pneumococcus* was more likely to be clindamycin‐resistant with penicillin sensitive; amoxicillin should be the first choice for ARI. The unnecessary use of cephalosporin and clindamycin may be related to the prescribing habits of doctors. A previous study found 26.6% of RTI patients in China were positive for *Mycoplasma pneumoniae* ( *M. pneumoniae* ), which was higher than the global incidence (12%) [29]. Azithromycin prescribed by physicians accounted for nearly one‐third of the antibiotic use in our study, and 11 patients who tested positive for RSV and/or influenza were prescribed azithromycin. A POCT for *M. pneumoniae* could help with the appropriate use of azithromycin.

There was a significant increased use in anti‐influenza drugs due to the positive detection rate of Flu B, which was consistent with existing research. Early treatment with antiviral drugs is recommended to relieve clinical symptoms and reduce the complications and mortality rate [30, 31]. However, the overuse of anti‐influenza drugs was also shown in our study, including patients who were given oseltamivir without medical advice and flu‐negative patients who received oseltamivir therapy. Since antimicrobial resistance has become a global public health threat, more education for the public about the appropriate use of antibiotics and antivirals is needed. Meanwhile, doctors should focus more attention on evidence‐based prescribing practices to decrease the unnecessary use of antibiotics [32].

We found that a higher virus detection rate can result in a decrease in antibiotic prescriptions. Therefore, we believe diagnostics are a useful tool to support the appropriate use of antibiotics. However, the use of rapid diagnostic tests to identify viral pathogens would not eliminate all prescriptions for antibiotics. Through logistic regression analysis, we found that Chinese doctors rely on the results of pathogen detection and consider the duration of fever when determining whether or not to prescribe antibiotics. It is generally believed that children with prolonged fever are at risk for complications due to bacterial infection. In the future, the use of bacterial biomarkers could help to specify which patients may require antibiotic treatment. In addition, if rapid diagnostic tests for bacterial infection were widely available, they could also help clinicians by providing pathogen‐specific information, thereby reducing the reliance on empirical judgment.

Our study has several limitations. First, our study is open‐label, and the two pediatricians who made treatment decisions may have differences in treatment practices. Second, the results of subgroup analysis, which compared antibiotics use before and after visit, had limitations due to the small sample size. Third, the cost of the Xpert test was not included in the total fee as it is not commercially available in China. Thus, the actual expenditure including the cost of Xpert testing could be higher. In addition, we did not test for RSV in the control group, and this could introduce some bias in the rate of antibiotic prescriptions.

In conclusion, the results of our study show that the Xpert Xpress Flu/RSV test can help to reduce the prescription of antibiotics for viral RTIs. Widespread use of rapid molecular diagnostic methods for ARI contributes to some extent to optimize management and improve antibiotic stewardship for children.

## Author Contributions

**Yue Xie:** writing – original draft, formal analysis, validation. **Tianming Chen:** formal analysis, writing – review and editing. **Bing Liu:** methodology. **Haijuan Xiao:** methodology. **Xinghui Gao:** supervision. **Qinjing Li:** methodology. **Bing Hu:** formal analysis, resources. **Cuiying Liu:** methodology. **Chengsong Zhao:** project administration, resources. **Yuchuan Li:** project administration, resources. **Xin Xu:** data curation, software. **Mengran Li:** formal analysis. **Yi‐Wei Tang:** writing – review and editing, conceptualization, funding acquisition. **Gang Liu:** conceptualization, writing – review and editing, funding acquisition.

## Funding

This work was supported by the Capital's Funds for Health Improvement and Research (2024‐1‐2092), Beijing Municipal Administration of Hospitals Incubating Program (PX2024042), and the Cepheid Investigator‐Initiated Study award (Cepheid‐IIS‐2020‐0003).

## Ethics Statement

This study was reviewed and approved by the Ethics Committee of Beijing Children's Hospital Affiliated to Capital Medical University ([2020]‐Y‐001).

## Consent

Written consent was obtained from the guardians of children.

## Conflicts of Interest

X.G., M.L., and Y.W.T. are employees of Cepheid/Danaher, the commercial manufacturer of the Xpert Xpress Flu/RSV. The other authors declare no conflicts of interest.

## Data Availability

All aggregated data are available in the manuscript. Individual participant data should be discussed with authors.
